# A colorimetric, photothermal, and fluorescent triple-mode CRISPR/cas biosensor for drug-resistance bacteria detection

**DOI:** 10.1186/s12951-023-02262-x

**Published:** 2023-12-20

**Authors:** Laibao Zheng, Yayun Jiang, Fuyuan Huang, Qiaoli Wu, Yongliang Lou

**Affiliations:** 1 Wenzhou Key Laboratory of Sanitary Microbiology, Key Laboratory of Laboratory Medicine, School of Laboratory Medicine and Life Sciences, Ministry of Education, Wenzhou Medical University, Wenzhou, Zhejiang China; 2 Department of Clinical Laboratory, People’s Hospital of Deyang City, Deyang, China

**Keywords:** CRISPR/Cas, Biosensor, Drug-resistance gene, Multimodal

## Abstract

A multimodal analytical strategy utilizing different modalities to cross-validate each other, can effectively minimize false positives or negatives and ensure the accuracy of detection results. Herein, we establish a colorimetric, photothermal, and fluorescent triple modal CRISPR/Cas12a detection platform (CPF-CRISPR). An MNPs-ssDNA-HRP signal probe is designed to act as a substrate to trigger three signal outputs. In the presence of the DNA target, MNPs-ssDNA-HRP is cleaved by the activated CRISPR/Cas12a, resulting in the release of HRP and generating short DNA strands with 3-terminal hydroxyl on magnetic beads. The released HRP subsequently catalyzed TMB-H_2_O_2_ reaction and oxidized TMB is used for colorimetric and photothermal signal detection. Under the catalysis of terminal deoxynucleotidyl transferase (TdT), the remaining short DNA strands are used as primers to form poly-T and function as scaffolds to form copper nanoclusters for fluorescent signal output. To verify the practical application of CPF-CRISPR, we employed MRSA as a model. The results demonstrate the platform’s high accuracy and sensitivity, with a limit of detection of 10^1^ CFU/mL when combined with recombinase polymerase amplification. Therefore, by harnessing the programmability of CRISPR/Cas12a, the biosensor has the potential to detect various drug-resistant bacteria, demonstrating significant practical applicability.

**Supplementary Information:**

The online version contains supplementary material available at 10.1186/s12951-023-02262-x.

## Introduction

The emergence of drug-resistant bacterial infections poses a significant threat to human health [1–3]. Traditional culture-based phenotype antimicrobial susceptibility testing (AST), while a gold standard, is time-consuming and costly, leading to delays in treatment [4]. This situation prompts physicians to rely on symptoms and experience for antibiotic decisions, potentially fueling misuse and resistance. Genotypic AST, as a complementary technique, offers several advantages in addressing these challenges. By detecting resistance genes, it provides a more rapid and targeted approach to determining the resistance status of microorganisms. *Staphylococcus aureus* (*S. aureus*) is a significant human pathogen, causing a wide range of infections from minor skin issues to life-threatening conditions like pneumonia and bloodstream infections. It is a leading cause of both hospital-acquired and community-acquired infections worldwide. Of particular concern is the increasing prevalence of methicillin-resistant *S. aureus* (MRSA), associated with more severe nosocomial and community-acquired infections. Rapid genotypic AST of MRSA through the detection of the mecA gene allows for quick identification and appropriate treatment decisions [5]. However, current gene detection techniques heavily rely on the polymerase chain reaction (PCR) and quantitative real-time PCR (qPCR), which demands sophisticated equipment and specialized expertise, thereby constraining its applicability, particularly in resource-limited settings [6]. Therefore, there is an urgent need to develop novel molecular diagnostic methods for drug resistance genes with high sensitivity, specificity, and adaptability for a broader range of detection settings.

Clustered regularly interspaced short palindromic repeats (CRISPR) and CRISPR-associated (Cas) systems are initially discovered in bacteria and archaea as an adaptive immune system to protect them from invading bacteriophages and viruses [7, 8]. Over time, the CRISPR/Cas system has become a transformative tool in the fields of gene editing [9, 10], cell imaging [11], and nucleic acid detection [12, 13] owing to its high identification of target genes. Notably, the found of CRISPR/Cas12a system which can indiscriminate cleavage single-stranded DNA (ssDNA) after the target-specific cleavage has highlighted the potential application of the CRISPR/Cas-based diagnostics such as DETECTR [14], and HOLMES [15]. To expand the utility of the CRISPR/Cas system to diverse scenarios, numerous signal output sensing strategies have been developed, such as colorimetric [16], electrochemical [17], SERS [18] and photothermal [19, 20]. However, the existing CRISPR/Cas biosensor mainly relied on a single signal output method which is susceptible to interference from various matrices, consequently impacting the accuracy of detection outcomes. Multiple signal output modes based detection method could improve the detection accuracy, and expand the detection range and application scenarios of the sensing strategy [21, 22].

To address these challenges, we developed a colorimetric, photothermal, and fluorescent triple signal-based CRISPR biosensor (CPF-CRISPR). This biosensor utilizes 3,3′,5,5′-tetramethylbenzidine (TMB) as a chromogen that can be oxidized by horseradish peroxidase (HRP) to produce oxidized TMB (oxTMB), resulting in a color change from colorless to blue and photothermal signal output under a near-infrared (NIR) laser irradiation. Additionally, fluorescent signals are generated using DNA-templated copper nanoclusters (CuNCs). Combined with the high sensitivity of fluorescent signals and easy acquisition of colorimetric and photothermal signals, the CPF-CRISPR method offers an extended detection range and increased accuracy, making it applicable in a broader range of scenarios. To verify the detection performance of the CPF-CRISPR platform, we selected MRSA as a drug-resistant model bacterium. The CPF-CRISPR platform showed satisfactory specificity and sensitivity with a limit of detection at 10^1^ CFU/mL for fluorescent signals. And, the practical application of the platform was verified by the isolation of *S. aureus* from clinical samples.

### Experimental section

#### Materials and reagents

Carboxyl-coated magnetic nanoparticles were purchased from PuriMag Biotechnology Co., Ltd. (Xiamen, China). Lysostaphin and oligonucleotides (Table S1) were obtained from Sangon Biotech Co. Ltd. (Shanghai, China). 2-(N- morpholino) ethane sulfonic acid (MES), TMB, 1-ethyl-3-[3-di-methylaminopropyl] carbodiimide hydrochloride (EDC), and 4-Morpholinepropanesulfonic acid (MOPS) were bought from Beijing Solarbio Biotechnology Co., Ltd. (Beijing, China). LbCas12a was purchased from New England Biolabs Inc. (United States). Terminal Deoxynucleotidyl Transferase (TdT) was bought from Takara Biotech Co., Ltd. (Dalian, China). Horseradish Peroxidase labeled streptavidin (SA/HRP) and DNase/RNase-free H_2_O were bought from Shanghai Beyotime Biotechnology Co., Ltd. (Shanghai, China). Copper sulfate (CuSO_4_·5H_2_O), Ascorbic acid (AA), and Sodium chloride (NaCl) were provided by Aladdin Biochemical Technology Co., Ltd. (Shanghai, China). RAAFAST, the recombinase polymerase amplification (RPA) nucleic acid amplification kits were obtained from Qitian Gene Biological Co., Ltd. (Jiangsu, China).

### Preparation of MNPs-ssDNA-HRP

Forty µL of magnetic beads (10 mg/mL) and 8 µL of 100 µM NH_2_-ssDNA-Biotin were added in 80 µL of MES Buffer (50 mM, pH 6.0). The mixture was incubated with shaking at room temperature for 30 min. Then, the 40 µL of the newly prepared 50 mg/mL EDC solution was added and shaken at room temperature for 4 h. The magnetic beads were washed with MES Buffer three times and isolated by magnetic decantation. 400 µL of 0.35 µg/mL SA/HRP was added in MNPs-ssDNA-Biotin and shaken at room temperature for 20 min. The MNPs-ssDNA-HRP were collected by magnetic separation and were washed with MES Buffer five times. Finally, to minimize background values, 400 µL of 0.5% BSA was added in MNPs, shaken at room temperature for 30 min, and washed 3 times with MES Buffer. Thus, MNPs-ssDNA-HRP is prepared.

### Measurement of the CPF-CRISPR

In our CRISPR/Cas12a activated cis-cleavage verification, the optimized conditions were utilized according to our previous reports [16, 23]. Briefly, 2 µL of the RPA amplification product was added into 4 µL of 1 µM Cas12a, 20 µL of 200 nM crRNA, 1 µg MNPs-ssDNA-HRP and 1 × NEBuffer r2.1 and incubated at 37 ℃ for 30 min. The solution was separated using magnetic separation. For the colorimetric assay, 10 µL of the reaction solution and 50 µL of TMB chromogen solution were mixed in the dark for 5 min. A color change from colorless (TMB) to bright blue (oxTMB) was observed due to the released HRP. 15 µL of H_2_SO_4_ (2 M) was added to terminate the reaction, and the changes in absorbance at 450 nm can be quantitatively measured using a microplate reader. Besides, due to its strong NIR laser-driven photothermal effect, oxTMB can be used for photothermal signal output. The oxTMB was irradiated for 2 min by 808 nm laser (5 W cm^− 2^) and the temperature of the solution was measured by a portable infrared imager. For the fluorescent assay, 5 µL Reaction Buffer (5×), 3 µL dTTP (100 mM), 1 µL TdT (10 U/µl), 2 µL BSA (0.1%), 14 µL H_2_O were added into MNPs and incubated at 37 ℃ for 1 h. The MNPs were washed with MES Buffer three times and isolated by magnetic decantation. Finally, 7 µL of 80 mM AA, 3.5 µL of 0.8 mM CuSO_4_, and 31.5 µL of MOPS Buffer (pH = 7.5) were mixed. The mixed solution was added to MNPs and the fluorescence intensity under excitation light at 340 nm was recorded. A BioTek Synergy Neo2 Microplate Reader NEO2S (Agilent, USA) was used to scan fluorescence and absorbance. A HC110 Dry Block Heater (DLAB, China) was applied to perform the RPA experiment. A FLIR E6 Handheld Infrared Camera (FLIR Systems, USA) was used to measure temperature changes. The 808 nm NIR laser was purchased from Changchun Laser Optoelectronics Technology Co., Ltd.

### Drug resistance gene detection

Twelve clinical strains including 8 strains of MRSA and 4 strains of methicillin-sensitive *S. aureus* (MSSA) were obtained from the People’s Hospital of Deyang City and approved by the Medical Research Ethics Committee. The 12 clinical *Staphylococcus aureus* had been validated for their resistance to oxacillin by the Vitek 2 system using the oxacillin MIC method. The detailed resistance information of *Staphylococcus aureus* to oxacillin is shown in Table S2. Bacterial Culture and preparation were optimized following the experimental protocol we used previously [16]. A single colony was cultured in Luria Bertani (LB) broth medium with shaking at 250 rpm for 8–12 h. Then the bacteria were washed three times at 3,500 rpm for 10 min with phosphate-buffered saline (PBS). The bacterial genome DNA was extracted using the boiling lysis method. Briefly, colonies were serially diluted to a concentration of 10^5^ CFU/mL, and the supernatant was removed by centrifugation. Next, 40 µL of 20 µg/mL lysostaphin was added to the precipitate and incubated at 37℃ for 15 min. The mixture was then incubated in boiling water for 5 minutes, and the resulting lysate was used for PRA using the RPA kit following the operation manual. Subsequently, the CPF-CRISPR platform is utilized for 12 clinical strains and 5 standard strain detection.

## Results and discussion

### Principle of the CPF-CRISPR biosensor

**Fig. 1:**
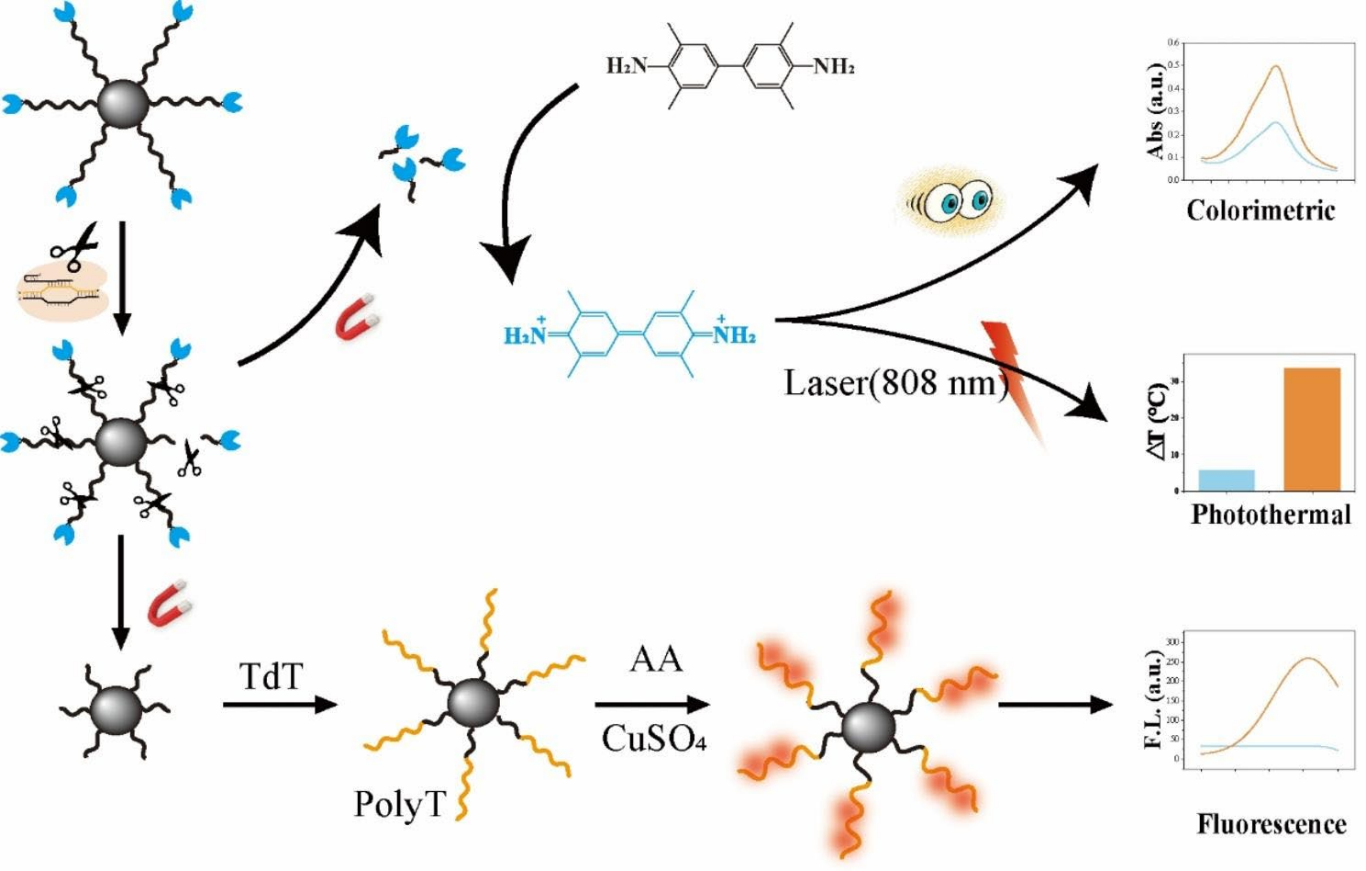
Schematic Illustration of the CPF-CRISPR Biosensor

As illustrated in Fig. 1, the 5’amino and 3’biotin-modified single-stranded DNA is designed to construct the MNPs-ssDNA-HRP signal probe, which is achieved by attaching the carboxyl magnetic beads and avidin-modified HRP enzyme to the DNA probe respectively. In the absence of target DNA, the MNPs-HRP signaling molecule remains intact and there is no corresponding signal output. In the presence of target DNA, the CRISPR/Cas12a system is activated, and the MNPs-ssDNA-HRP is indiscriminately catalytically cleaved by Cas12a, resulting in the HRP being released. After magnetic separation, the released HRP catalyzes the TMB to oxTMB for colorimetric detection by the naked eye. Notably, oxTMB, as a strong photothermal agent, has a strong photothermal conversion effect under 808 nm laser irradiation, leading to an increase in the temperature of the solution [24]. CuNCs have the advantages of abundant precursor and high fluorescence performance which are more favorable for fluorescent applications [25]. Compared with other bases, the T base has a higher affinity for copper to form CuNCs and the fluorescence intensity correlates with the length of Poly-T [26]. Terminal deoxynucleotidyl transferase (TdT) is a unique enzyme that catalyzes the synthesis of Poly-T by the addition of dTTPs to the 3-terminal hydroxyl end of primers [27]. Interesting, non-specific cleavage of MNPs-HRP signaling probe by Cas12a leads to the generation of new 3-terminal hydroxyl moieties on magnetic beads. Using the new 3-terminal hydroxyl acting as a template, a TdT-mediated isothermal amplification system was initiated and generated a poly-T tail. In the presence of Cu^2+^ and AA, these poly-T subsequently function as scaffolds for the formation of CuNCs, leading to fluorescence signal output.

### Validation of the CPF-CRISPR biosensor

A series of experiments were conducted to validate each component of the CPF-CRISPR detection platform. Figure S1 revealed that the targets specifically triggered the trans-cleavage of the CRISPR/Cas12a system by fluorescence probe. The successful synthesis of the MNPs-ssDNA-HRP probe was also verified. As shown in Figure S2, the absorbance at 450 nm was significantly higher in the MNPs-ssDNA-HRP group which indicated the successful conjugation between the magnetic bead and SA/HRP by a ssDNA. Sequentially, since thymine (T) has a high affinity for Cu^0^ and can promote the reduction of copper ions to copper monomers, we used the synthesized poly-T as a template to verify the feasibility of CuNCs preparation. As the fluorescence spectra of CuNCs shown formed by Poly-T, the CuNCs exhibited strong emission peaks at 654 nm with an excitation wavelength of 340 nm (Figure S3). Finally, polyacrylamide gel electrophoresis reveals the generation of Poly-T by TdT on magnetic beads (Figure S4).

**Fig. 2:**
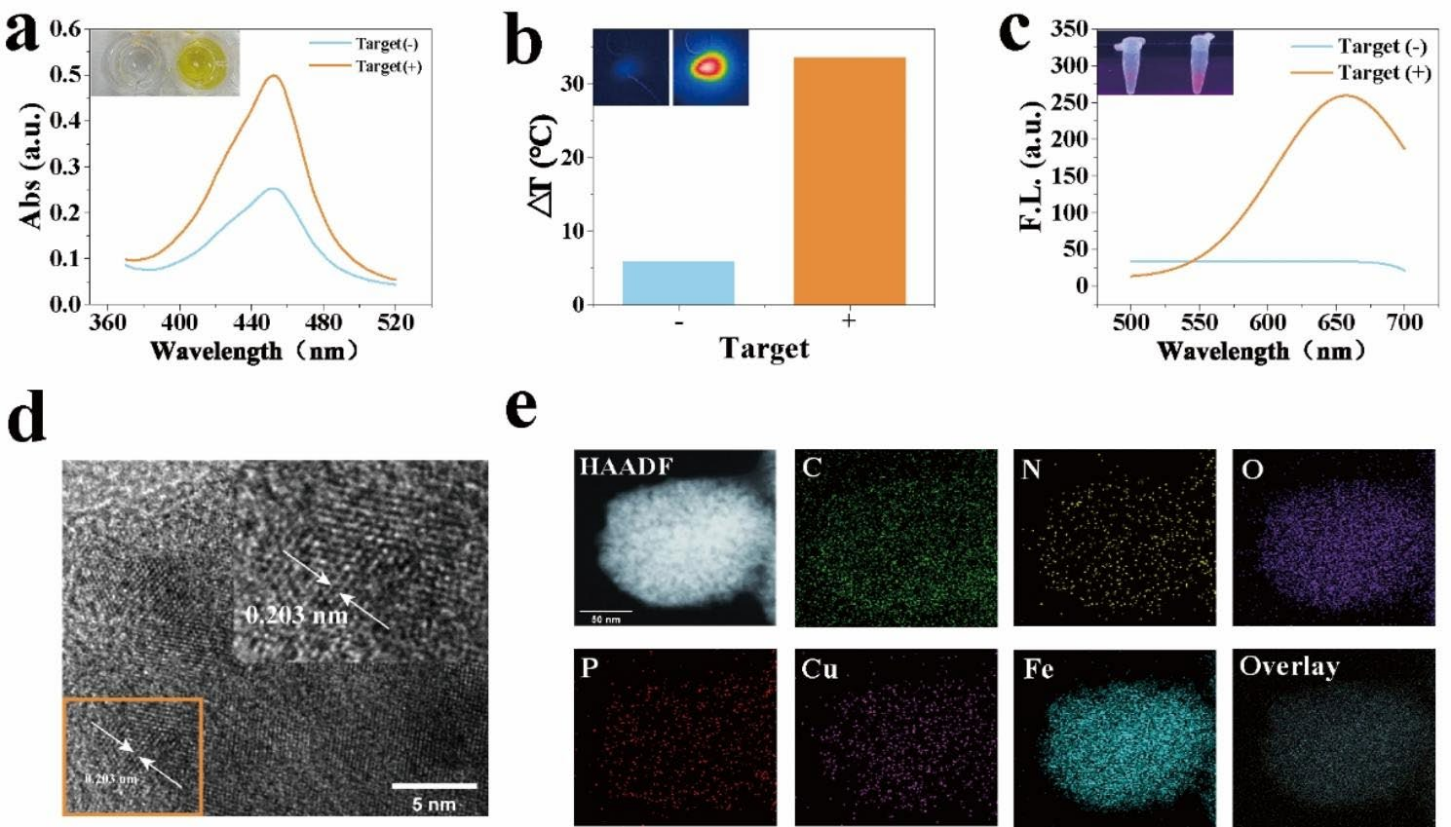
Feasibility analysis of CPF-CRISPR Platform. (**a**) Absorption spectra against the target by CPF-CRISPR platform. (**b**) The feasibility of photothermal signal outputs by CPF-CRISPR platform. ΔT (℃) = T_1_- T_0_, T1 is the temperature after laser irradiation, and T_0_ is the initial temperature. (**c**) The feasibility of fluorescence signal outputs by CPF-CRISPR platform. (**d**) TEM image of copper nanoclusters on the surface of magnetic beads. (**e**) HRTEM and EDS-mapping image of nanoclusters on the surface of magnetic beads. The visual observation results of colorimetry, photothermal, and fluorescent were listed above the corresponding figure

The feasibility of tri-modal signal outputs of the CPF-CRISPR biosensor was verified sequentially. For colorimetric detection, in the presence of the target, the endonuclease activity of CRISPR/Cas12a is activated and HRP is released from magnetic beads. The released HRP catalyzed the TMB to oxTMB with a color change from colorless to blue (Fig. 2a). With strong near-infrared laser-driven, oxTMB has a photothermal effect which is wide applications in photothermal signal output. As shown in Fig. 2b, the temperature of the Target (+) group was significantly increased after laser irradiation at 808 nm. For fluorescence detection, under the catalysis of TdT, the poly-T is generated as a primer to form and functions as scaffolds to form CuNPs to give a high signal (Fig. 2c). The TEM was used to observe the morphology and size of the generated CuNCs on magnetic beads (Figure S5). As shown in Fig. 2d, The CuNCs deposited on the surface of magnetic beads appeared, and the average diameter was about 0.203 nm. EDS analysis of element composition showed the presence of C, N, O, P, Fe, and Cu (Figure S6), which also proved the formation of copper nanoclusters on the surface of the beads. Element mapping of CuNCs shows All elements are evenly distributed on the surface of magnetic beads (Fig. 2e).

### Optimization of experimental conditions

**Fig. 3:**
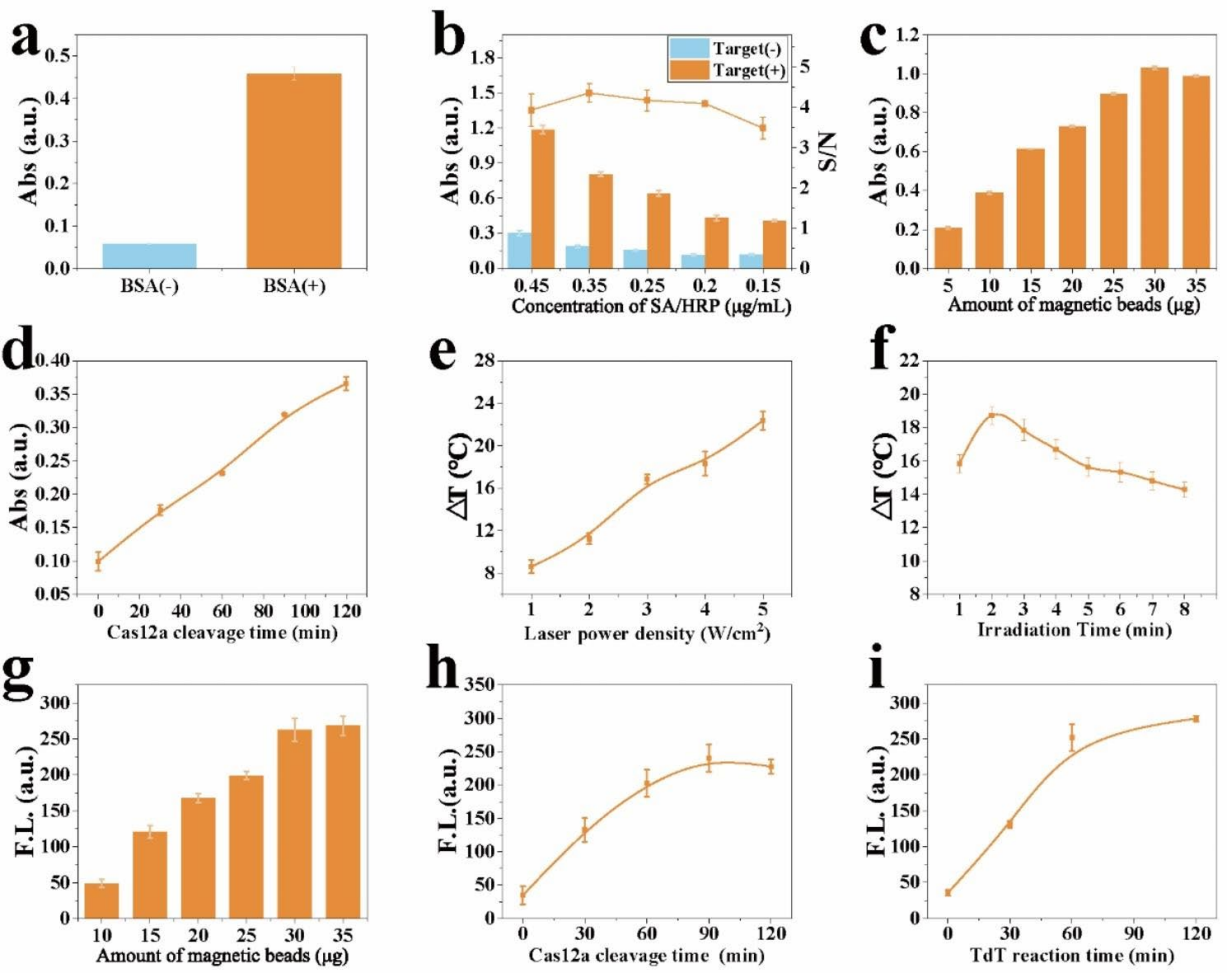
Optimization of experimental conditions. (**a**) The effects of BSA encapsulation on absorbance. (**b**) The concentration of HRP on absorbance. (**c**) The amounts of magnetic beads on absorbance. (**d**) The trans-cleavage time on absorbance. (**e**) the laser power density on ΔT of the platform. (**f**) the irradiation time on ΔT of the platform. ΔT (℃) = ΔT (Target+)- ΔT (Target-), ΔT (Target+) is the temperature change after laser irradiation upon targets, ΔT (Target-) is the temperature change after laser irradiation upon no target. (**g**) The trans-cleavage time on fluorescent intensity. (**h**) The TdT reaction time on fluorescent intensity. (**i**) The usage of magnetic beads on fluorescent intensity

To obtain the excellent detection performance of the CPF-CRISPR system, the experimental conditions for each of the three signal output methods were optimized. The effects of BSA encapsulation, HRP concentration, dosage of magnetic bead, and the trans-cleavage time of Cas12a are the key factors affecting the oxTMB signaling molecules. As shown in Fig. 3a, the 0.5% BSA-encapsulated magnetic bead can significantly increase the absorbance when target genes are present. This may be due to the encapsulation of BSA, which attenuates the non-specific adsorption of the magnetic beads to the free HRP produced after cutting. To improve the TMB color changes, the concentration of HRP was investigated ranging from 0.15 µg/mL to 0.45 µg/mL. 0.35 µg/mL with the highest signal-to-noise ratio was selected as the best concentration in subsequent research (Fig. 3b). As shown in Fig. 3c, the dosage of magnetic beads is explored by the absorbance at 450 nm over 5 to 35 µg, and the absorbance reached a peak at 30 µg. The absorbance at 450 nm is linearly related to the Cas12a cleavage time from 0 to 120 min (Fig. 3d). The power of the laser irradiation and the irradiation time have an important influence on the performance of the photothermal signal. As shown in Fig. 3e, the temperature rapidly increases with the increment of the laser power density from 1 W/cm^2^ to 5 W/cm^2^. Therefore, 5 W/cm^2^ is selected as the optimal laser power density. As the laser irradiation time increases, the temperature change appears to increase and then decrease. Therefore, the peak value of 2 minutes was selected for further experiments (Fig. 3f). The amounts of magnetic beads used and Cas12a cleavage time not only affect the colorimetric signal but also play an important role in the fluorescence signal (Fig. 3g, h). To obtain optimal signal output, the final dosage of magnetic beads at 30 µg and Cas12a cleavage time at 60 min was used in the CPF-CRISPR platform. The TdT reaction time is a key element that limits the performance of fluorescent signals, and the fluorescence intensity had nearly plateaued at 60 min and was selected for a follow-up experiment (Fig. 3i).

### Analytical performance of the CPF-CRISPR platform

**Fig. 4:**
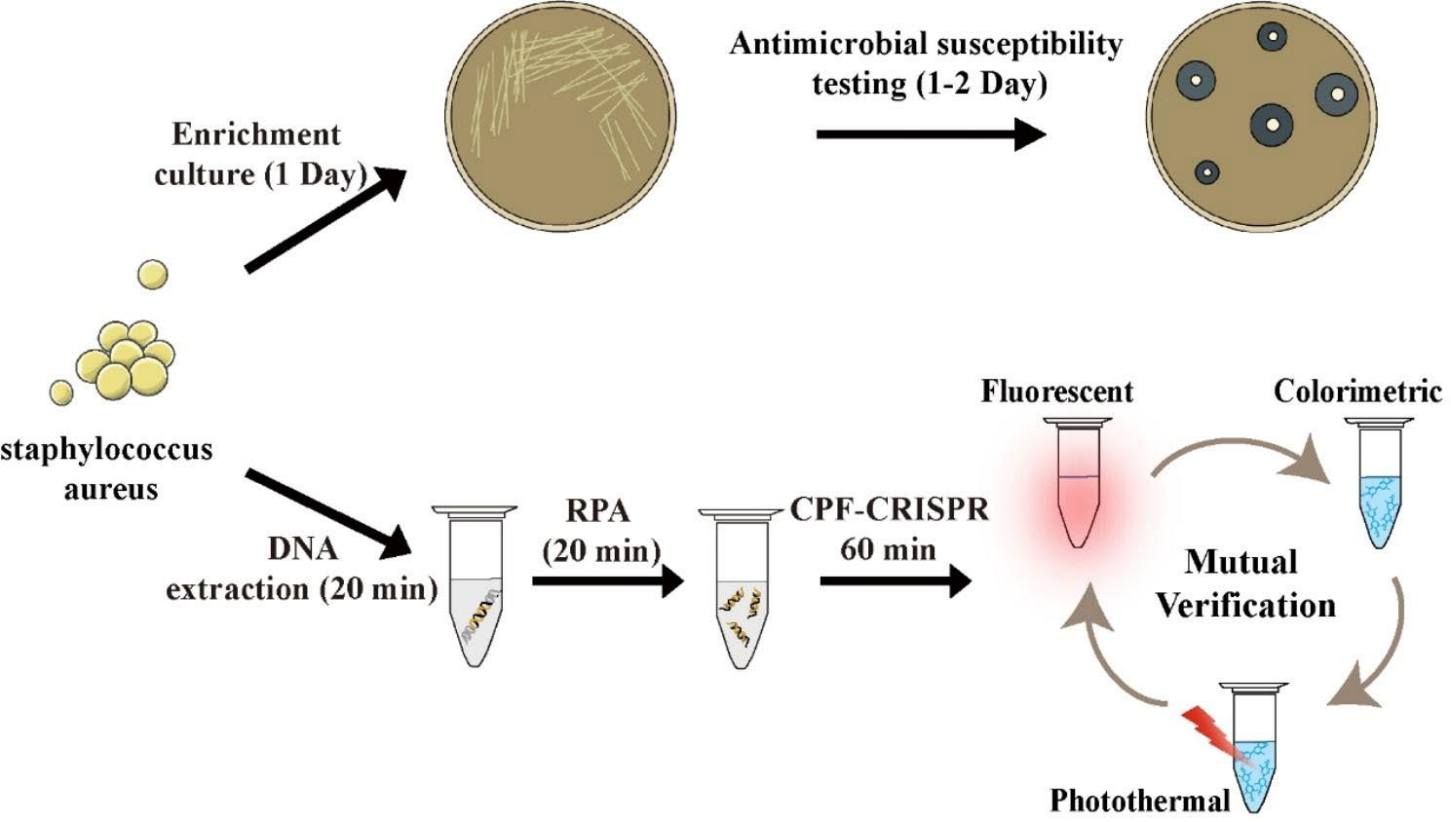
Schematic illustration of the CPF-CRISPR platform and culture-based standardized MRSA assays

MRSA is a drug-resistant bacterium of focus in hospitals, and its traditional culture-based standardized assays need 2–3 days [28]. The mecA gene, which encodes the low-affinity penicillin-binding protein 2a that leads to methicillin resistance in staphylococci, is used as a MRSA-specific gene for nucleic acid diagnosis [27, 29]. Herein, we combined CPF-CRISPR with RPA (Figure S7) and validated the fluorescent, colorimetric, and photothermal signal outputs of the CPF-CRISPR Platform with MRSA (ATCC 43,300) as the detection object, respectively (Fig. 4). To maximize the detection performance of CPF-CRISPR, RPA technology was employed to amplify the detection target, and the amplification time of RPA has been optimized. As shown in Figure S8, the time of RPA for the colorimetric signal is 30 min and 15 min for the fluorescence signal for the detection of 10 CFU/mL of MRSA.

**Fig. 5:**
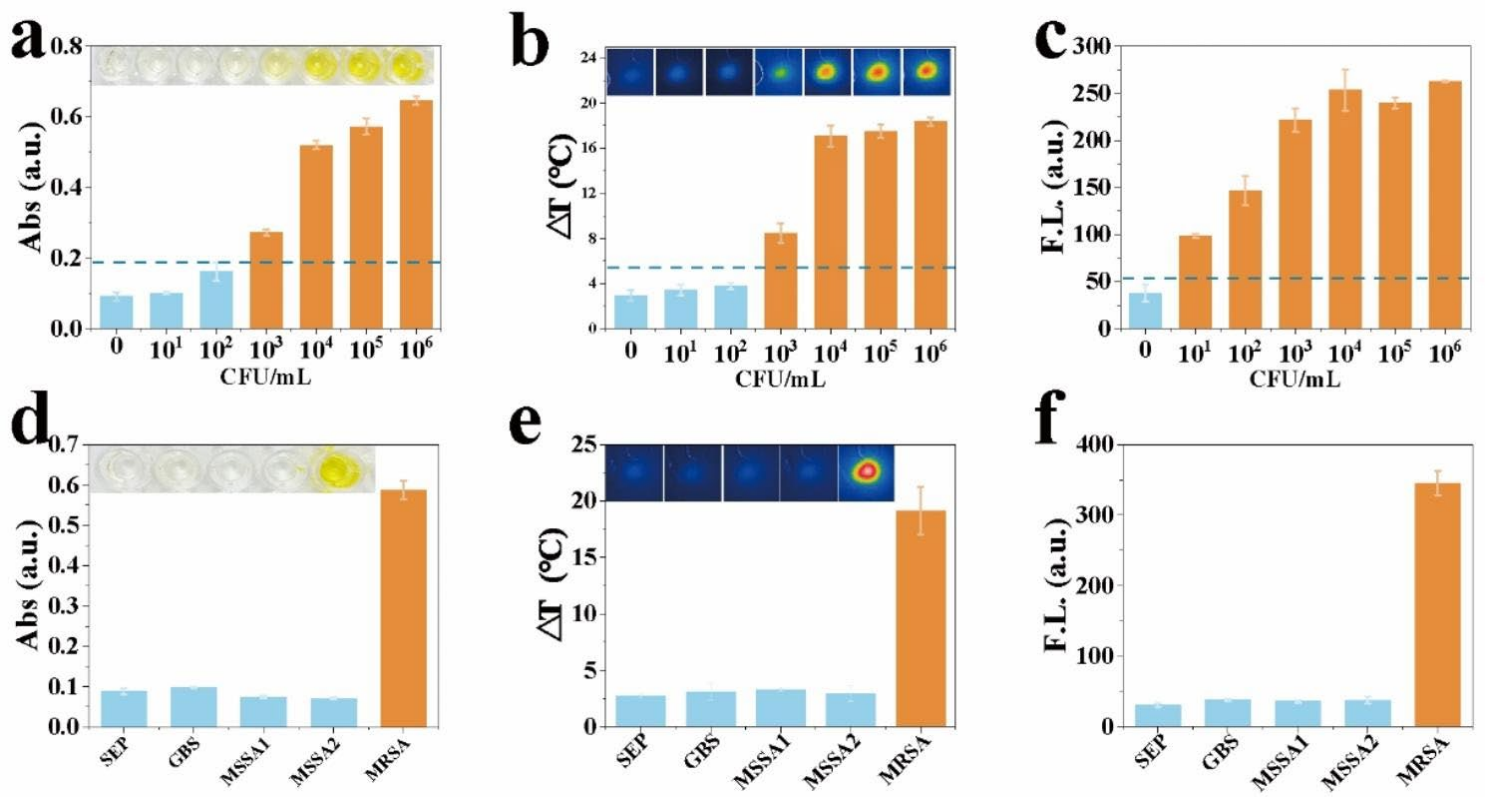
Analytical Performance of the CPF-CRISPR Platform. (**a**) absorbance, (**b**) temperature change, and (**c**) fluorescent intensity of the CPF-CRISPR platform for the measurement of different concentrations of MRSA ranging from 10^1^ CFU/mL to 10^6^ CFU/mL. (**d**) absorbance, (**e**) temperature change, and (**f**) fluorescent intensity of the CPF-CRISPR platform for the measurement of MRSA (ATCC 43,300) and two MSSA (ATCC 29,213, ATCC 25,923) and two Gram-positive cocci (*Staphylococcus epidermidis* and *Streptococcus agalactiae Group*). Error bars represent mean ± SD, where n = 3 replicates. The visual observation results of colorimetry and photothermal were listed above the corresponding photos

We evaluated the sensitivity and specificity of the CPF-CRISPR system for the detection of MRSA under optimal conditions. To verify the cut-off value of the CPF-CRISPR platform, we performed multiple replicates without using MRSA (Figure S9). The results of the CPF-CRISPR platform against different concentrations of MRSA showed that the three signal strengths increased correspondingly with the number of colony reductions from 0 CFU/mL to 10^6^ CFU/mL (Fig. 5a-c). The selectivity of the CFP-CRISPR platform for MRSA (ATCC 43,300) was investigated with two MSSA (ATCC 29,213, ATCC 25,923) and two Gram-positive cocci (*Staphylococcus epidermidis* and *Streptococcus agalactiae Group*). To ensure the accuracy of the results, the expression of mecA in five bacterial strains was first verified by agarose gel electrophoresis (Figure S10). The MRSA (ATCC 43,300) induced obvious changes in absorption, temperature, and fluorescence intensity, while the other four strains had little signal intensity changes (Fig. 5d-f), indicating good selectivity of this platform.

**Fig. 6:**
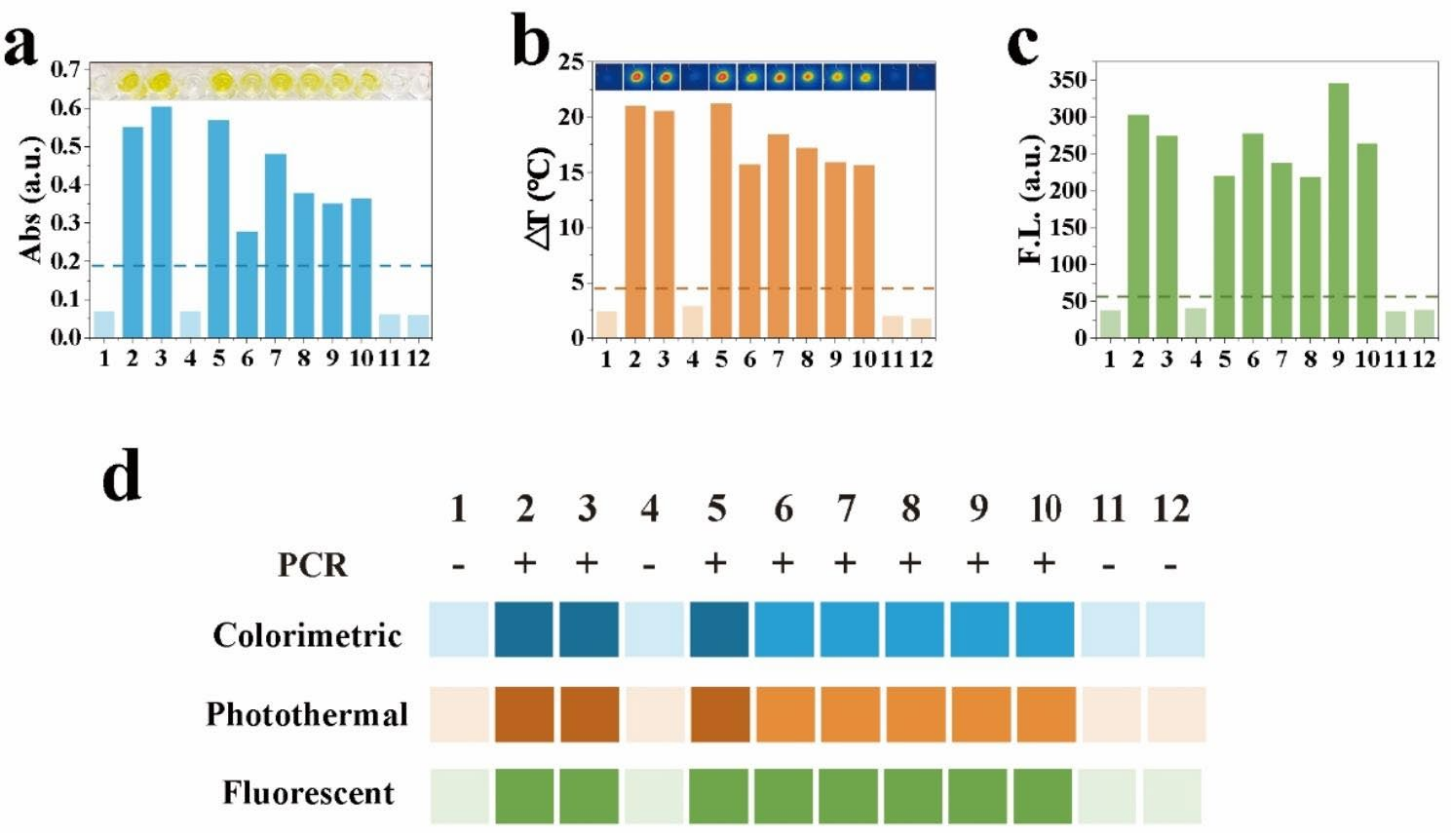
Clinically isolated strain analysis using the CPF-CRISPR. Clinical isolated strain analysis by (**a**) colorimetry, (**b**) photothermal, and (**c**) fluorescence. The visual observation results of colorimetry and photothermal were listed above the corresponding figure

To further validate the performance of the CPF-CRISPR platform in clinical sample applications, we collected 12 clinical isolates of *S. aureus*, including 8 strains of MRSA and 4 strains of MSSA. All isolated strains were verified for methicillin resistance by clinical drug sensitivity assay (Table S2) and the presence of mecA was verified by PCR using agarose gel electrophoresis (Figure S11). All eight MRSA strains displayed noticeable colorimetric, photothermal, and fluorescent signal changes, while the MSSA strains exhibited only extremely weak signal fluctuations and were below the threshold level (Fig. 6a-c). Besides, As shown in Fig. 6d, the colorimetric, photothermal, and fluorescent results were in perfect agreement, further confirming the reliability of the results. These results further demonstrate that the CPF-CRISPR assay platform has a good detection performance. So, the CPF-CRISPR platform holds great potential for the detection of drug-resistant bacteria in real applications.

## Conclusions

In summary, we have developed a triple-modal detection method incorporating colorimetric, photothermal, and fluorescent signal outputs by harnessing the trans-cleavage activity of CRISPR/Cas12a, the TMB catalytic reaction, and the fluorescence property of copper nanocluster. Rigorous optimization of experimental conditions has been undertaken to ensure the efficient performance of these three signal methods, enabling easy visual detection, portability, and enhanced sensitivity across various scenarios. Moreover, the system provides signal cross-validation, enhancing accuracy and reliability. Using the MRSA as a model, we have demonstrated the method’s high sensitivity and specificity, making the CFP-CRISPR system a promising platform for detecting drug-resistant bacteria. This innovation holds the potential to make a significant impact on medical diagnostics, environmental monitoring, and food safety, particularly in resource-constrained settings.

### Electronic supplementary material

Below is the link to the electronic supplementary material.

Supplementary Material 1

## Data Availability

All data generated or analyzed during this study are included in the article.
