# Supplementary material for: A colorimetric, photothermal, and fluorescent triple-mode CRISPR/cas biosensor for drug-resistance bacteria detection
Source: J Nanobiotechnology. 2023 Dec 20;21:493. doi: 10.1186/s12951-023-02262-x (PMC10731848; doi:10.1186/s12951-023-02262-x)
Supplement: Supplementary file 1 — Supplementary Material 1 [file 12951_2023_2262_MOESM1_ESM.docx]

**A Colorimetric, Photothermal, and Fluorescent Triple-Mode CRISPR/Cas Biosensor for Drug-Resistance Bacteria Detection**

**Laibao Zheng^a,†,*^, Yayun Jiang^b,†^, Fuyuan Huang^a^, Qiaoli Wu^a^, Yongliang Lou^a,*^**

^a^ Wenzhou Key Laboratory of Sanitary Microbiology, Key Laboratory of Laboratory Medicine, Ministry of Education, China, School of Laboratory Medicine and Life Sciences, Wenzhou Medical University, Wenzhou, Zhejiang, China

^b^ Department of Clinical Laboratory, People’s Hospital of Deyang City, Deyang, China

^*^Corresponding authors: zhenglaibao@wmu.edu.cn (Laibao Zheng); lyl@wmu.edu.cn (Yongliang Lou);

**^†^**Laibao Zheng and Yayun Jiang contributed equally to this work.

**Table S1.** The sequences of oligonucleotides used in this work.

| **Names** | **Sequences (5’-3’)** |
| --- | --- |
| NH_2_-DNA-Biotin | TTTTTTTTAGAAGAAGGTGTTTAAGTATTTTTTTTTTTTTTTTTTTTTTTTTTTTTTTT |
| Poly-T | TTTTTTTTTTTTTTTTTTTTTTTTTTTTTTTTTTTTTTTTTTTTTTTTTTT |
| crRNA | UAAUUUCUACUAAGUGUAGAUCUAGAGGAUAGUUACGACUU |
|  |  |
| RPA-F | TTCAACGCCACACGAGACTGGTGTAATGCGG |
| RPA-R | CCAATGTAAGTGCGGCGGTTTGCCCAACTCTGG |
| PCR-F | TGTTTATGGTGAGAACGGTGACA |
| PCR-R | TTCTTTATCTAGGCCCCAAACTTG |

**Table S2.** Clinical strain information and screening for methicillin resistance by Vitek 2 system using oxacillin MIC method.

| **Sample number** | **Original clinical**  **specimen** | **Vitek 2 MIC of**  **oxacillin ^a^** | **Detection results** |
| --- | --- | --- | --- |
| 1 | sputum | ≤0.25 | MSSA |
| 2 | sputum | ≥4 | MRSA |
| 3 | sputum | ≥4 | MRSA |
| 4 | secretin | 0.5 | MSSA |
| 5 | secretin | ≥4 | MRSA |
| 6 | sputum | ≥4 | MRSA |
| 7 | secretin | ≥4 | MRSA |
| 8 | secretin | ≥4 | MRSA |
| 9 | blood | ≥4 | MRSA |
| 10 | blood | ≥4 | MRSA |
| 11 | secretin | ≤0.25 | MSSA |
| 12 | secretin | 0.5 | MSSA |

^a^ The determination of methicillin resistance of clinically isolated *Staphylococcus aureus* is completed by the Vitek 2 system using oxacillin MIC method (oxacillin concentrations range from 0.25 to 4 μg/mL). According to the CLSI drug susceptibility standard, the *Staphylococcus* *aureus* with a MIC of oxacillin ≥4 μg/mL is MRSA, while MIC of oxacillin ≤2 μg/mL is MSSA.


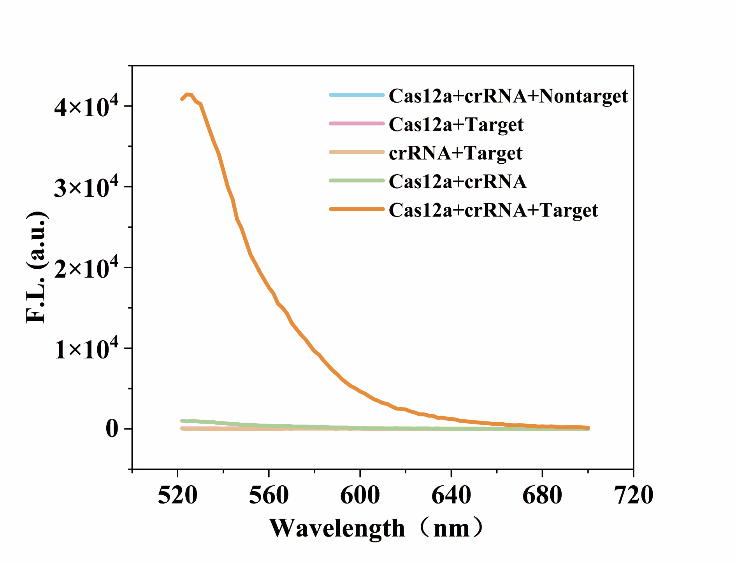


**Fig.S1.** Feasibility assessment by fluorescent analysis to confirm that the trans-cleavage of CRISPR-Cas12a system can be activated in the presence of target DNA.


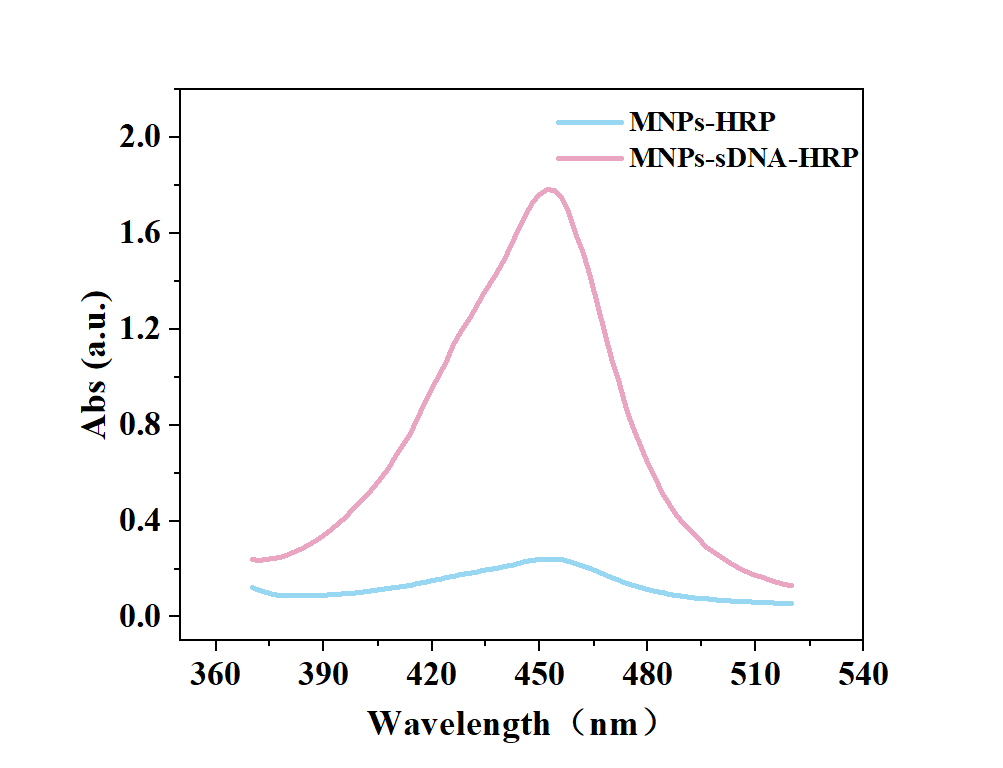


**Fig.S2.** Absorption spectra for MNP-sDNA-HRP signal probe catalyzed TMB-H_2_O_2_ reaction.


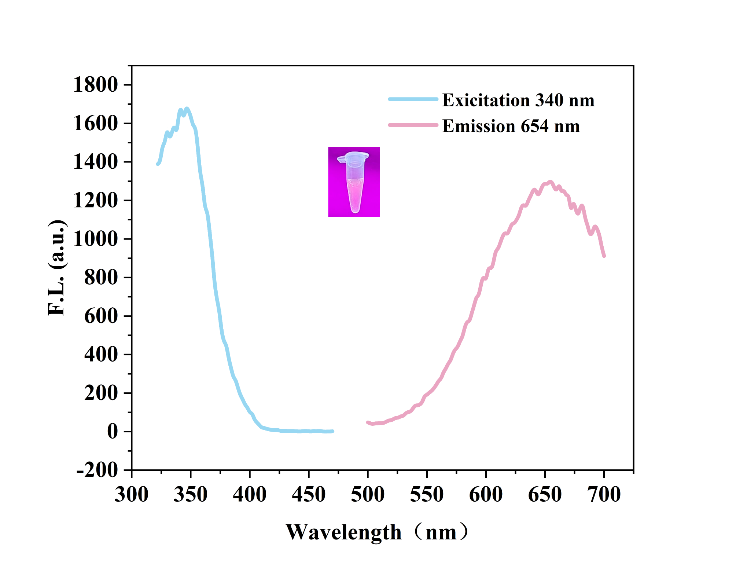


**Fig.S3.** Fluorescence spectrum of CuNCs. The inset is the image of CuNCs illuminated under UV light.


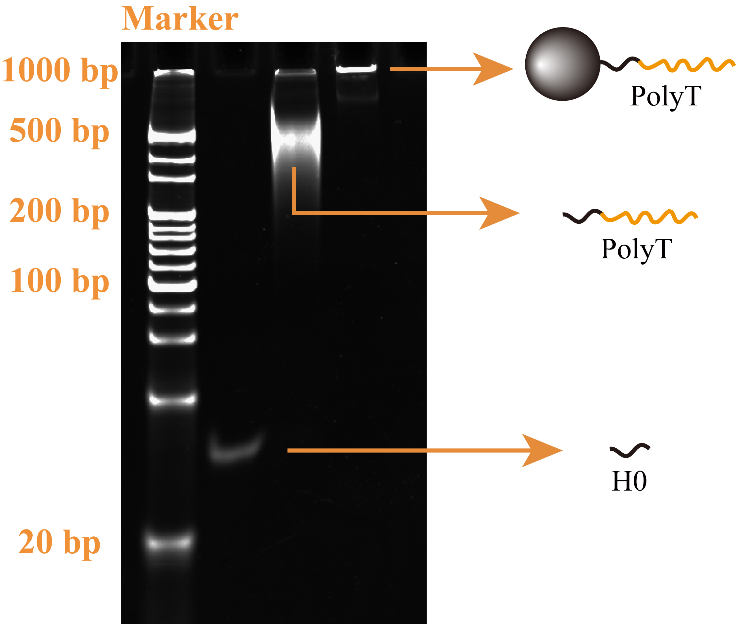


**Fig.S4.** PAGE analysis of feasibility of PolyT formation on magnetic beads.


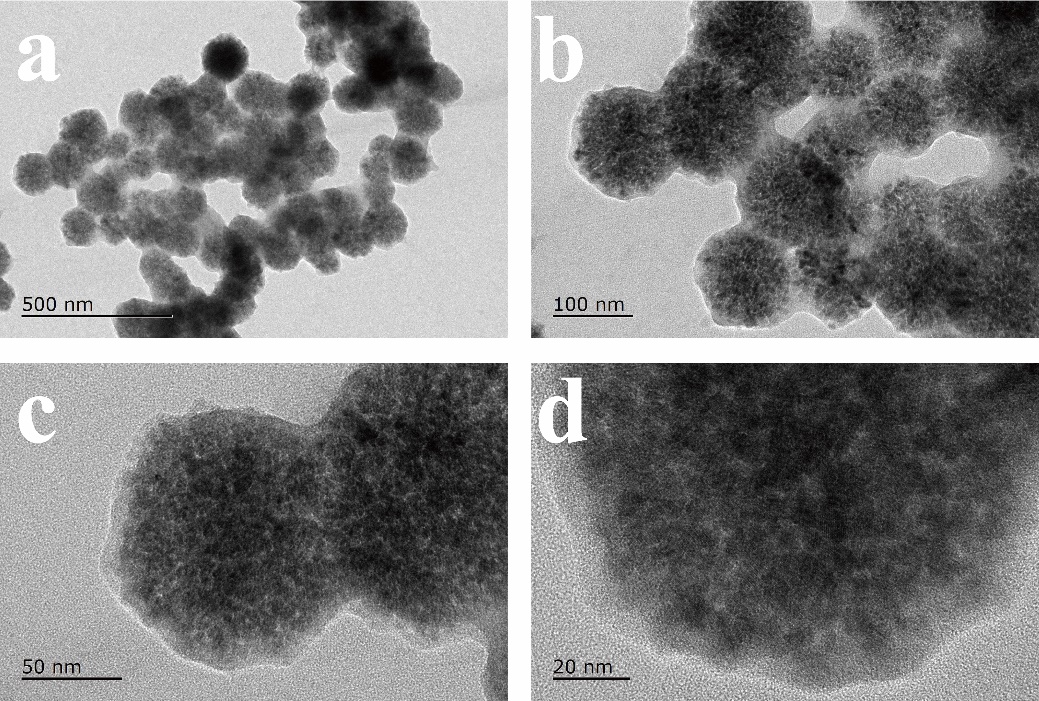


**Fig.S5.** TEM images of CuNCs on the magnetic beads


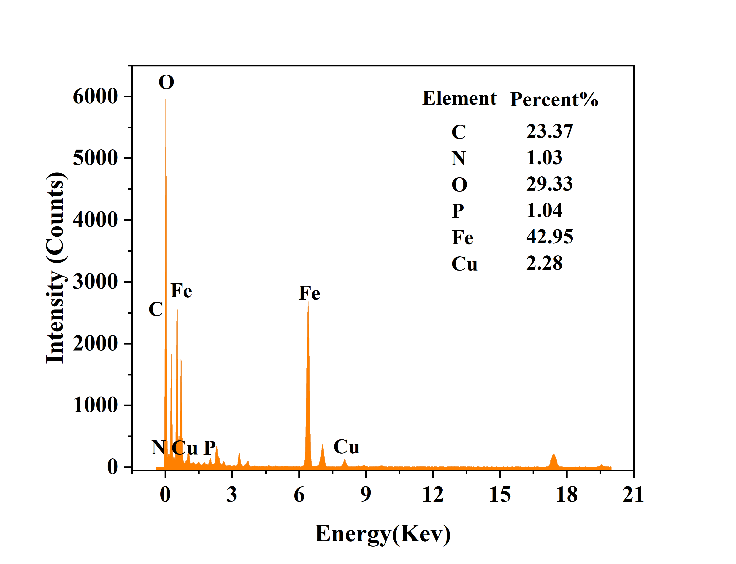


**Fig.S6.** EDS pattern of CuNCs on the magnetic beads.


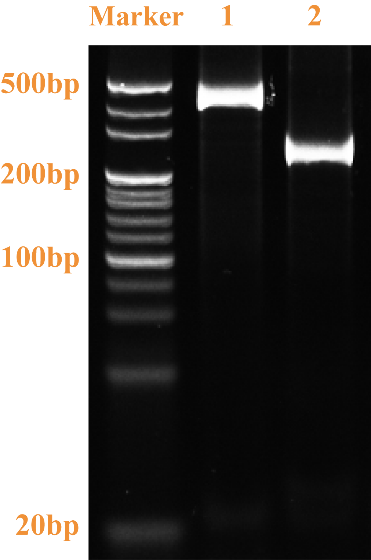


**Fig.S7.** PAGE analysis of amplification of the mecA gene of MRSA(ATCC43300) by RPA with different primer set. Lanes 1 and 2 correspond to RPA amplification of the mecA gene by primer set 1 and 2.
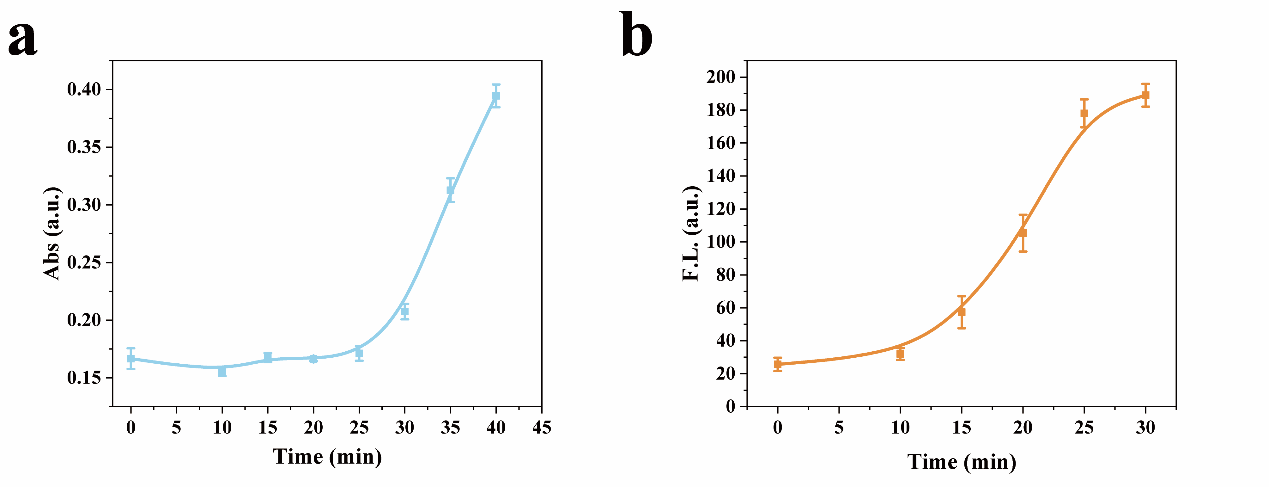


**Fig.S8.** (a) The RPA amplification time on the absorbance of the CPF-CRISPR. (b) The RPA amplification time on the fluorescence intensity of the CPF-CRISPR.


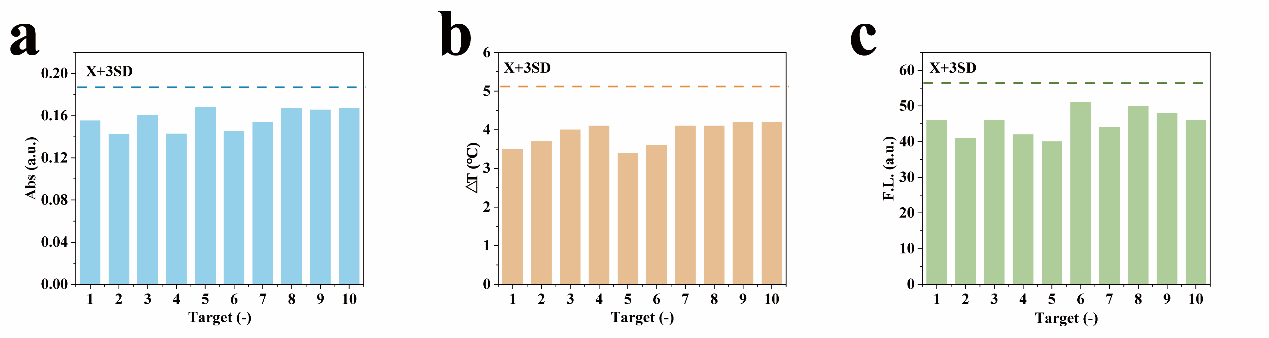


**Fig.S9.** Calculate the cut-off value for colorimetric, photothermal, and fluorescent outputs of RCRISPR-CPF platforms. the cut-off value is calculated by X + 3SD, X represents an average signal value of three replicates without using the target gene, and SD represents three standard deviations.


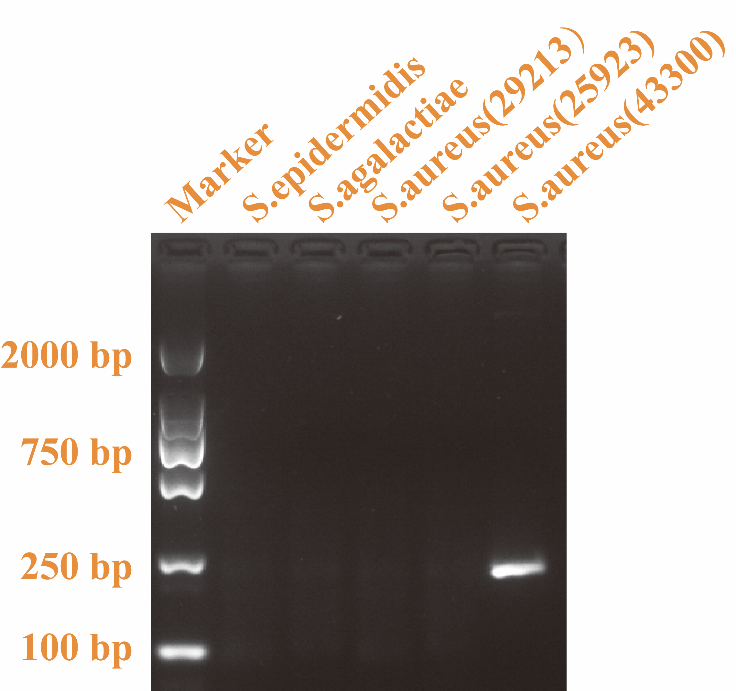


**Fig.S10.** Analysis of mecA gene expression in three *Staphylococcus aureus* and two Gram-positive cocci by 3% agarose gel electrophoresis


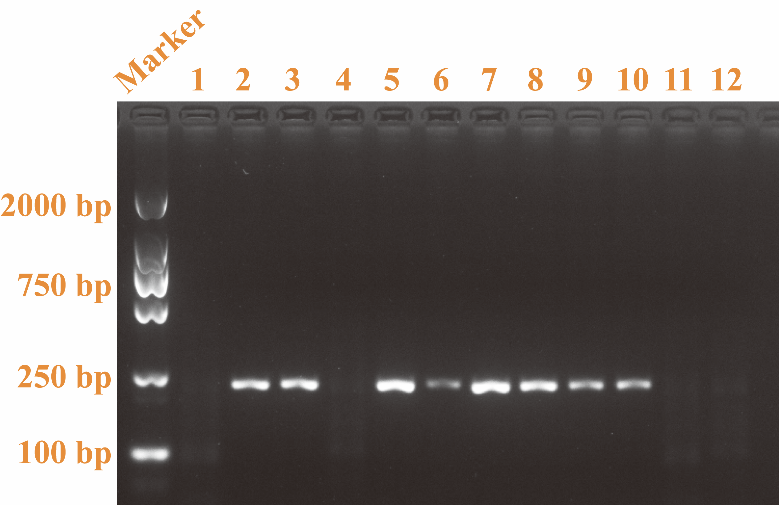


**Fig.S11.** Analysis of mecA gene expression in clinical isolates strains by 3% agarose gel electrophoresis
